# Enzymatic Stoichiometry Reveals the Metabolic Limitations of Soil Microbes under Nitrogen and Phosphorus Addition in Chinese Fir Plantations

**DOI:** 10.3390/microorganisms12081716

**Published:** 2024-08-20

**Authors:** Yan Ren, Ying Wang, Xiulan Zhang, Xionghui Liu, Pei Liu, Liang Chen

**Affiliations:** 1 Faculty of Life Science and Technology, Central South University of Forestry and Technology, Changsha 410004, China; 2 Huitong National Station for Scientific Observation and Research of Chinese Fir Plantation Ecosystems in Hunan Province, Huitong 438107, China; 3 Key Laboratory of Forest Ecology and Management, Institute of Applied Ecology, Chinese Academy of Sciences, Shenyang 110016, China; 4 National Engineering Laboratory for Applied Technology of Forestry & Ecology in South China, Changsha 410004, China

**Keywords:** nitrogen deposition, phosphorus addition, soil enzymes’ stoichiometry, Chinese fir, microbial nutrient limitations

## Abstract

Increasing nitrogen (N) deposition alters the availability of soil nutrients and is likely to intensify phosphorus (P) limitations, especially in P-limited tropical and subtropical forests. Soil microorganisms play vital roles in carbon (C) and nutrient cycling, but it is unclear whether and how much N and P imbalances affect the soil’s microbial metabolism and mechanisms of nutrient limitations. In this study, a 3-year field experiment of N and P addition (control (CK), 100 kg N ha^−1^ yr^−1^ (N), 50 kg P ha^−1^ yr^−1^ (P), and NP) was set up to analyze the extracellular enzyme activities and stoichiometry characteristics of the top mineral soils in Chinese fir plantations with different stand ages (7, 20, and 33 years old). The results showed that the enzyme activities associated with the acquisition of C (β-1,4-glucosidase (BG) and β-d-cellobiohydrolase (CBH)) and P (acid phosphatases (APs)) in the N treatment were significantly higher than those in the CK treatment. Moreover, vector analysis revealed that both the vector’s length and angle increased in stands of all ages, which indicated that N addition aggravated microbial C and P limitations. The P and NP treatments both significantly decreased the activity of AP and the enzymes’ N:P ratio, thereby alleviating microbial P limitations, as revealed by the reduction in the vector’s angle. Stand age was found to promote all enzymatic activities but had no obvious effects on the limitation of microbial metabolism with or without added nutrients in the soils under Chinese fir. Available N, Olsen-P, and pH were the main drivers of microbial metabolic limitations related to C nutrients. These results provide useful data for understanding the change in soil microbial activity in response to environmental changes, and suggest that P fertilization should be considered for management to improve productivity and C sequestration in Chinese fir plantation in the context of increased deposition of N.

## 1. Introduction

Forests’ productivity and carbon (C) sequestration are widely constrained by the availability of soil nitrogen (N) and phosphorus (P) [1,2]. In the past few decades, the deposition of atmospheric N has increased in forest ecosystems and is expected to increase further in the future [3,4]. However, anthropogenic P inputs have increased to a much smaller extent [5]. As a consequence, stoichiometric imbalances in soil C, N, and P are expected to be initiated or aggravated, which can alter plant productivity and biogeochemical processes [5,6]. Soil microorganisms play an important role in the decomposition of organic C, nutrient cycling, and tree growth [7]. Extracellular enzyme activities (EEAs), as key indicators of microbial nutrient acquisition and metabolic processes, can describe the soil’s C turnover and nutrient release [8,9]. Therefore, exploring the dynamics of soil EEAs and enzymatic stoichiometry in response to N and P inputs will provide deep insights into the mechanism of C nutrient cycling in microorganisms, which will be important for forest management in the context of global N deposition.

Extracellular enzymes secreted by soil microorganisms are directly involved in the degradation of high-molecular-weight organic matter and nutrient release [10]. β-1,4-glucosidase (BG), β-d-cellobiohydrolase (CBH), β-1,4-N-acetylglucosaminidase (NAG), leucine aminopeptidase (LAP), and acid or alkaline phosphatases (AP) have been widely used as indicators of microbial demand for energy (C) and nutrients (N and P) since they catalyze terminal reactions that produce assimilable molecules containing C, N, and P from high-molecular-weight organic compounds [11]. Sinsabaugh et al. [12] suggested that the relative activities of (BG + CBH)/(NAG + LAP) and (BG + CBH)/AP reflect the relative demand for acquiring C versus N and C versus P, respectively. Moreover, Moorhead et al. [13] proposed calculating the length and angle of the vectors of enzymatic stoichiometry to quantify the relative C versus nutrient limitations and relative P versus N limitations of soil microorganisms, respectively. Within this context, the activities of extracellular enzymes in the soil and their stoichiometric ratios are closely related to changes in the availability of soil nutrients [6,14]. Numerous studies on the effects of adding nutrients on soil EEAs have shown that this can increase or decrease enzymes for the acquisition of C, N, and P to some extent, and that different enzymes do not respond consistently to the addition of nutrients [15,16,17]. For instance, meta-analyses have shown that N addition generally increased the activities of enzymes for the acquisition of P [18] and hydrolases for the acquisition of C [19]. By contrast, other meta-analyses reported that N addition has minor or negative effects on N-acquisition enzymes [8,20]. Meanwhile, P addition generally decreases the activity of P-acquiring enzymes and vector angles; i.e., it can alleviate microbial P limitations [6,21]. Many studies have also analyzed the enzymatic stoichiometry among C-, N-, and P-acquiring enzymes; in these studies, the enzymes’ C:P and N:P ratios increased with the availability of P [22], while the enzymatic C:N ratio was positively correlated with available N in the soil [8]. These results highlight our uncertainty about the regulation of soil EEAs and enzymatic stoichiometry in the context of a changing soil nutrient status. It was also noted that soil enzymes’ response to nutrient addition is highly context-dependent and varies with environmental factors [23]. Therefore, further research is needed to determine how forest soils’ microbial resource limitations change under the scenario of increased N or P inputs.

Chinese fir (*Cunninghamia lanceolata* [Lamb.] Hook), a fast-growing evergreen coniferous tree species with high yield and good wood quality, has been widely planted in southern China for timber production [24]. The total planting area has reached 9.9 million ha and accounts for about 12.4% of the total plantation area in China [25]. However, due to successive rotation, these plantations suffer from a series of management problems, including declining soil fertility and the deterioration of the soil’s physicochemical properties [26]. Meanwhile, global deposition of N has led to soil N accumulating over time and the aggravation of P limitations in subtropical soil, which has become one of the main factors limiting high productivity in Chinese fir plantations [17,27]. Because soil microbes and their extracellular enzymes play an important role in the decomposition of organic matter and the release of nutrients, the microbiological mechanisms underlying the response of plantations to soil nutrient imbalances need to be further explored. It should be noted that the impact of N and P inputs on EEAs may differ across stands of different ages due to plant–microbial interactions [6]. During the development of Chinese fir, the tree’s biomass typically increases in parallel with an increase in nutrient uptake [28]. Meanwhile, litterfall increases as stands develop, which may favor decomposers that produce hydrolytic enzymes [29]. Thus, understanding the responses of limitations in soil microbes’ metabolism to nutrient addition and the stand’s age will help to create reasonable management practices for maintaining productivity.

In this study, a 3-year experiment on N and P addition was conducted in Chinese fir plantations of different ages (7, 20, and 33 years) to determine how soil EEAs and enzymatic stoichiometry respond to changes in the availability of soil nutrients induced by ambient N and P inputs. The main objectives of this study were to (i) reveal the effects of adding N and P on the characteristics of the metabolic limitations of soil microbes across different-aged stands of Chinese fir; (ii) decipher the main driving factors of microbial metabolic limitations in the soils of Chinese fir plantations in response to N and P inputs. We hypothesized that N addition would increase soil N availability and possibly aggravate microbial C or P limitations, associated with a shift to a higher investment in C- or P-acquiring enzymes, while P addition would alleviate microbial P limitations and result in in fewer resources being allocated to P-acquisition enzymes. Moreover, we expected that N addition’s aggravation of P limitation would be more obvious in the mature stands than in the young stands.

## 2. Materials and Methods

### 2.1. Study Sites and Experimental Design

This research was conducted in Huitong County (26°41′50″–26°47′08″ N, 109°35′26″–109°38′45″ E), southwest of Hunan Province, China, which has an average annual temperature of 16.8 °C and an average annual rainfall of 1268 mm. This region has a typical humid subtropical monsoon climate, and the soil type is Alliti-Udic Ferrosols developed from shale parent rock [30]. The native vegetation in the mountains of this region has been almost replaced with Chinese fir for timber production. Currently, clear-cut harvesting in Chinese fir plantations has generated a patchwork of stands of various ages.

In August 2018, we selected stands of three different ages planted in 2014 (4 years old; young), 2001 (17 years old; middle-aged), and 1988 (30 years old; mature), according to the local management agency. All of the selected Chinese fir plantations were second-generation with the same management practices. To minimize the differences in the climate and parent soil materials, these stands were within 2.0 km of each other to guarantee that differences among the stands were predominantly caused by age. Moreover, all the stands had similar topography, altitude, and soil texture, and were all distributed on well-drained uplands with a mean altitude of about 400 m and slopes ranging from 20° to 30° [30]. Understory shrubs in the plantations consisted of *Smilax china*, *Maesa japonica*, and *Ilex purpurea*, and herbs included *Dicranopteris linearis*, *Woodwardia japonica*, and *Cyclosorus parasiticus*. The average contents of Fe, Al, and Mn in the soil of the Chinese fir plantations were about 32 g kg^−1^, 75 g kg^−1^, and 0.95 g kg^−1^, respectively. In each stand, 16 plots measuring 10 m × 10 m were randomly set up, with four each for the control (no N or P was added, CK), N addition (100 kg N hm^−2^ a^−1^, N), P addition (50 kg P hm^−2^ a^−1^, P), and the co-addition of N and P (100 kg N hm^−2^ a^−1^ + 50 kg P hm^2^ a^−1^, NP), respectively. Urea (CO(NH_2_)_2_) was applied as the N treatment, and sodium dihydrogen phosphate (NaH_2_PO_4_) was applied as the P treatment, both by spraying of an aqueous solution. Fertilizer was applied to the soil’s surface four times per year from 2018 using backpack sprayers. Specifically, N and P application in the growing season (March and June) accounted for 60% of the total annual application, while application outside the growing season (September and December) accounted for 40%. At each fertilization, the corresponding dose of solute was weighed and then fully dissolved in 6 L of deionized water. The same amount of water was applied to control plots to avoid the effect of additional water. Buffer strips of over 5 m were set up between the plots.

### 2.2. Soil Sampling and Preparation

Soil samples were collected in July 2021. After removal of the surface litter, the top layer of mineral soils (0–10 cm) was collected at five points (one point at the center and four points equidistant from the center toward the corners of the subplots) of each plot. The fresh soil samples were then transported to the laboratory and sieved through a 2 mm mesh to remove coarse-grained materials before being analyzed further. One set of soil subsamples was air-dried and sieved in preparation for the physicochemical analyses of C, N, and P content, and another set was stored at 4 °C for the determination of inorganic N and microbial enzyme activity. Additional subsamples were stored at −80 °C for analyses of the microbial community.

### 2.3. Analysis of the Soil’s Physicochemical Properties

The soil moisture content was measured by oven-drying the fresh soil samples at 105 °C for 24 h. Soil pH was measured at a soil-to-water ratio of 1:2.5 with a pH meter. Soil organic carbon (SOC) was measured using the K_2_Cr_2_O_7_ oxidation method [31]. Semi-micro Kjeldahl digestion using CuSO_4_, K_2_SO_4_, and Se as the catalysts was performed to determine the soil’s total N (TN). Soil-available N (the sum of ammonium N (NH_4_^+^-N) and nitrate N (NO_3_^−^-N)) was extracted from the soils with 2 M KCl solution (soil: solution = 1:4) and measured using a continuous flow analyzer (AA3, Bran + Lubbe, Norderstedt, Germany). Total P (TP) was measured by the molybdate/ascorbic acid method after H_2_SO_4_-HClO_4_ digestion. Olsen-P was determined by a method using 0.05 mol L^−1^ HCl-0.025 mol L^−1^ (1/2 H_2_SO_4_) [32]. The C:N ratio was calculated from the SOC and TN content, the C:P ratio was calculated from the SOC and TP content, and the N:P ratio was calculated from the TN and TP content.

### 2.4. Measurements of Phospholipid Fatty Acids (PLFAs) in the Soil

Phospholipid fatty acids (PLFAs) were analyzed to characterize the structure of the soil microbial community [33]. Total lipids were extracted from samples of 3 g (dry weight) of soil using 20 mL of a single-phase extraction reagent (chloroform:methanol:citric acid = 1:2:0.8). The phospholipids were converted to fatty acid methyl esters (FAMEs), which were analyzed via gas chromatography (HP 7890 series; Agilent Technologies Inc., Santa Clara, CA, USA), and the concentrations of FAMEs were calculated using a microbial ID system (MIDI INo-C., Netwark, DE, USA). We used MIDI peak identification software (Version 4.5) to identify individual PLFAs. Each fatty acid was quantified by comparing the individual peak area with that of the internal standard 19:00, and its unit was nmol PLFA g^−1^ dry soil. For this, i15:0, a15:0, i16:0, a16:0, 16:0, 16:1ω5, 16:1ω7, 16:1ω9, i17:0, a17:0, 17:0, cy17:0, 10Me17:0, 18:0, 18:1ω7, 18:1ω9, 10Me18:0, 18:2ω6, cy19:0, and 20:0 characterized total PLFAs; i14:0, i15:0, a15:0, i16:0, i17:0, a17:0, 16:1ω9c, 16:1ω7c, cy17:0, 18:1ω7c, cy19:0, 10Me16:0, 10Me17:0, and 10Me 18:0 characterized bacteria; 18:1ω9c and 18:2 ω6c characterized fungi; 16:1ω5c characterized mycorrhizal fungi; i15:0, a15:0, i16:0, i17:0, and a17:0 characterized Gram-positive bacteria (G^+^); and 16:1ω7c, cy17:0, 18:1ω7, and cy19:0 characterized Gram-negative bacteria (G^−^). The G^+^:G^−^ and F:B ratios were calculated as the ratio of Gram-positive to Gram-negative bacterial PLFAs and the ratio of fungal to bacterial PLFAs, respectively.

### 2.5. Soil Enzymatic Activity Analyses

The activity of the soil extracellular enzymes, including BG, CBH, NAG, LAP, and AP, was measured by the 96-microtiter enzyme plate fluorescence assay [34]. In brief, a soil suspension was made by adding 1 g of fresh soil to 125 mL of a 50 mM sodium acetate buffer (pH = 5.3) and mixing them with a magnetic mixer. Next, 0.2 mL of the soil suspension was pipetted into a 96-well microtiter plate and 50 μL of a 200 mM substrate was added, and all wells were incubated for 4 h at 25 °C in the dark. After the incubation had been stopped, 10 μL of a 1.0 M NaOH solution was added to each well and, after 1 min, fluorescence was measured using a microplate fluorometer (Synergy H4, BioTek, Winooski, VT, USA) using 365 nm excitation and 450 nm emission filters. Eight replicates were set up for each sample, along with a blank, a soil control, a substrate control, and a standard curve. After correcting for negative controls and quenching, enzymes’ activities were expressed in units of nmol g^−1^ h^−1^ dry soil.

### 2.6. Vector Analysis of Resource Limitations of Soil Microbes

Vector analysis (vector length (VL, unitless) and vector angle (VA, degrees)) of soil enzyme activity was conducted to evaluate the limitations of soil microbial C and nutrient metabolism [13,35]. Vector length was calculated as the square root of the sum of x^2^ and y^2^, and vector angle was calculated as the arctangent of the line extending from the plot origin to point (x, y), where x represents the relative activity of C- versus P-acquiring enzymes (i.e., (BG + CBH)/(BG + CBH + AP)) and y represents the relative activity of C- versus N-acquiring enzymes (i.e., (BG + CBH)/(BG + CBH + NAG + LAP)) [36,37]. The lengths of the vectors represent relative C vs. nutrient limitations and vector angles represent relative P vs. N limitations. Longer vectors indicate greater C limitations. Vectors with angles of <45° and >45° indicate the relative degree of N limitation and P limitation, respectively. Vector lengths and angles were calculated as follows:

Vector length = SQRT (x^2^ + y^2^)

Vector angle (o) = Degrees (Atan2 (x, y))

The enzymes’ C:N, C:P, and N:P ratios were calculated as follows [38]:

Enzyme C:N = *ln* (BG + CBH): *ln* (LAP + NAG)

Enzyme C:P = *ln* (BG + CBH): *ln* (AP)

Enzyme N:P = *ln* (LAP + NAG): *ln* (AP)

### 2.7. Statistical Analysis

Data analysis was performed using SPSS (version 20.0; SPSS Inc. Chicago, IL, USA). One-way analysis of variance (ANOVA) was used to investigate changes in the soil’s physicochemical properties, the enzymes’ activity, and the enzymes’ stoichiometry ratios under different treatments and stand ages. Differences were considered significant at *p* < 0.05 via a post hoc least significant difference (LSD) test. Spearman’s correlation, followed by post hoc tests and the Mantel test, were used to investigate the relationships among VL, VA, the soil’s properties, and the microbial community’s structure. A redundancy analysis (RDA) was used to identify the most significant factors that affected soil enzyme activities and the enzymes’ stoichiometry ratios. All figures were drawn using Origin Pro 2019 (Origin Lab Corporation).

## 3. Results

### 3.1. Changes in Soil Properties and Microbial Structure

**Figure 1:**
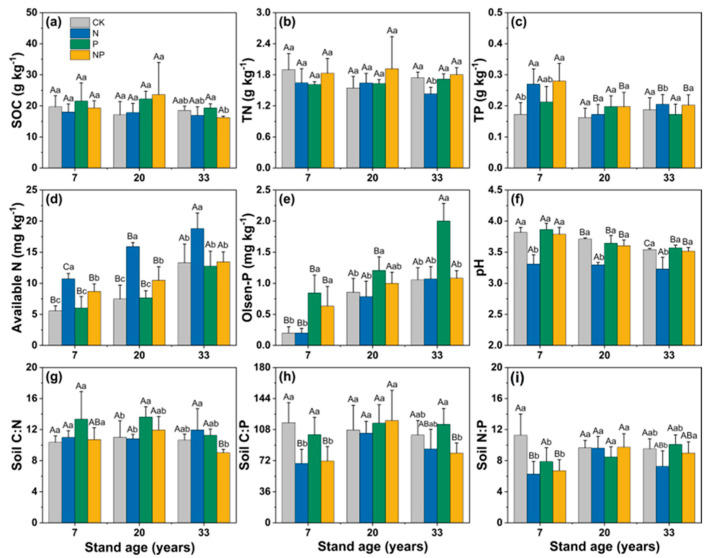
Effects of adding N and P on the soil’s properties. (**a**) SOC, soil organic carbon; (**b**) TN, total nitrogen; (**c**) TP, total phosphorus; (**d**) available N, the sum of ammonium N (NH_4_^+^-N) and nitrate N (NO_3_^−^-N); (**e**) Olsen-P; (**f**) pH; (**g**) soil C:N, ratio of SOC to TN; (**h**) soil C:P, ratio of SOC to TP; (**i**) soil N:P, ratio of TN to TP. Different lowercase letters indicate significant differences (*p* < 0.05) among different treatments in stands of the same age, and different uppercase letters indicate significant difference among different stand ages.

SOC, TN, and TP contents did not show a significant change across the stands of different ages with or without the addition of nutrients (*p* > 0.05, Figure 1a–c). However, the soil-available N (the sum of NH_4_^+^-N and NO_3_^−^-N) was significantly increased (average: 72%) under the addition of N, and the Olsen-P content significantly increased (47%) under the addition of P in all three stand ages (*p* < 0.05, Figure 1d,e). The co-addition of N and P significantly increased the soil-available N or Olsen-P content in the young and middle-aged stands, but not in the mature stand (Figure 1d,e). N addition also significantly reduced soil pH (2.5%), while P and NP treatments had no effect on pH (*p* < 0.05, Figure 1f). P addition significantly reduced the soil N:P ratio in young stands (*p* < 0.05), and the NP treatment significantly reduced the soil C:P and N:P ratios in young stands (*p* < 0.05; Figure 1h,i).

**Figure 2:**
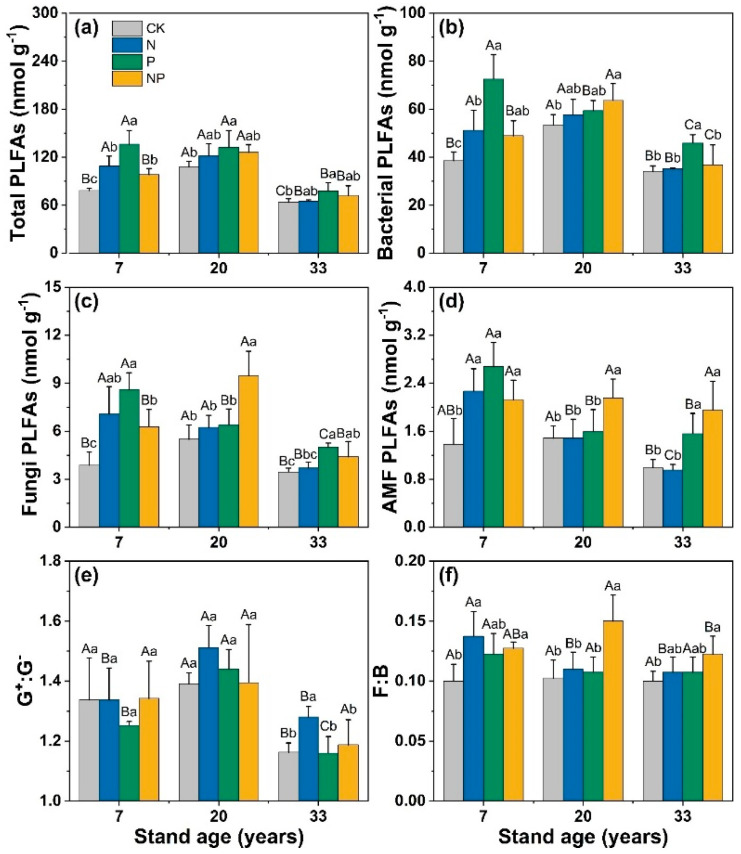
Effects of N and P addition on the soil’s microbial biomass and community structure (indicated by PLFAs) in Chinese fir plantations of different ages. The unit for microbial biomass is nmol g^−1^. (**a**) Total PLFAs; (**b**) Bacterial PLFAs; (**c**) Fungal PLFAs; (**d**) AMF PLFAs, arbuscular mycorrhizal fungal PLFAs; (**e**) G^+^:G^−^, the ratio of Gram-positive to Gram-negative bacterial PLFAs; and (**f**) F:B, the ratio of fungal to bacterial PLFAs. Different lowercase letters indicate significant differences (*p* < 0.05) among different treatments in stands of the same age, and different uppercase letters indicate significant differences among different stand ages.

N addition significantly increased the total PLFAs (57%), bacteria (38%), fungi (75%), AMF (77%), and F:B ratio (40%) in the 7-year-old stand (*p* < 0.05; Figure 2). P addition significantly increased total PLFAs (73%), bacteria (87%), and fungi (97%) in the 7-year-old stands, and significantly increased AMF in the 7-year-old (90%) and 33-year-old (23%) stands (*p* < 0.05). NP treatment significantly increased the total PLFAs (28%) of young stands, the bacteria and fungi in 7-year-old and 20-year-old stands, and AMF (average 68%) and the F:B ratio (42%) in stands of all ages (*p* < 0.05).

### 3.2. Changes in the Activity of Soil Extracellular Enzymes

**Figure 3:**
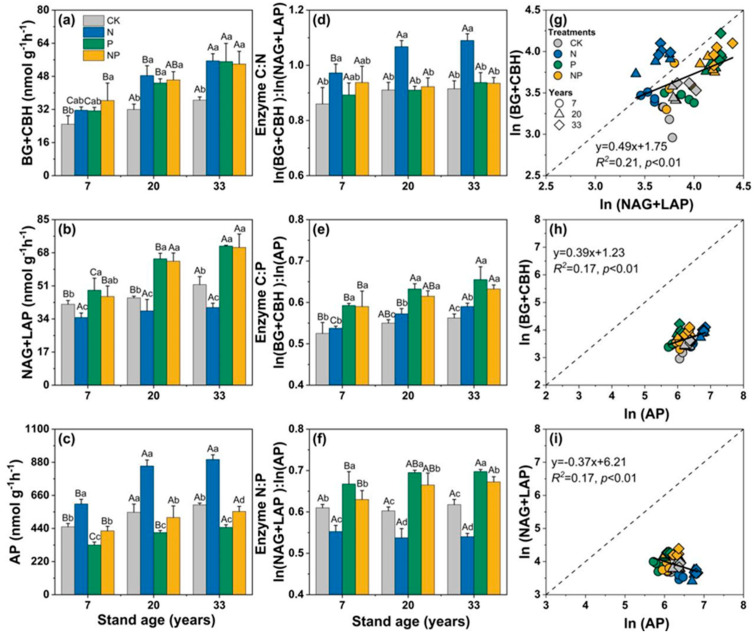
Effects of N and P addition on soil enzyme activity. (**a**–**c**) The stoichiometric ratio of soil enzymes (**d**–**f**), and standard major axis regressions between (**g**) log-transformed activities of NAG + LAP and BG + CBH, (**h**) log-transformed activities of AP and BG + CBH, and (**i**) activities of log-transformed AP and NAG + LAP. The slopes of all regressions were significant at *p* < 0.01. BG + CBH, sum of β-1,4-glucosidase and β-d-cellobiohydrolase; NAG + LAP, sum of β-1,4-N-acetylglucosaminidase and leucine aminopeptidase; AP, acid phosphatase. Different lowercase letters indicate significant differences (*p* < 0.05) among different treatments in stands of the same age, and different uppercase letters indicate significant differences among different stand ages.

After 3 years of controlled experiments, there were significant differences in the activity of soil C-acquiring (BG + CBH), N-acquiring (NAG + LAP), and P-acquiring (AP) enzymes and the stoichiometric ratios between the control and nutrient treatments (Figure 3). N addition significantly increased the activity of C (average: 46%)- and P (52%)-acquiring enzymes but decreased that of N (24%)-acquiring enzymes (*p* < 0.05, Figure 3), and these effects gradually increased with the stands’ age. P addition significantly increased C (45%)- and N (38%)-acquiring enzymes’ activities, especially in 20-year-old and 33-year-old stands, while significantly decreasing AP (37%) activity for all stand ages (*p* < 0.05, Figure 3). The co-addition of N and P also significantly increased C- and N-acquiring enzymes’ activities, but slightly decreased AP activity in all the stands (*p* < 0.05, Figure 3).

The ratios of the activity of BG + CBH, NAG + LAP, and AP showed the potential acquisition of C vs. N or P (Figure 3). The enzymes’ C:P and N:P ratios were less than 1 under the CK, N, P, and NP treatments. Compared with the control, the addition of N significantly increased the enzymes’ C:N ratio for all ages of stands and the enzymes’ C:P ratio for the 20-year-old and 33-year-old stands, while decreasing the enzymes’ N:P ratio for stands of all ages (*p* < 0.05). P addition significantly increased the enzymes’ C:P and N:P ratios for stands of all ages but had no effect on the C:N ratio. NP addition significantly increased the enzymes’ C:P ratio for all stands and the N:P ratio for the 20-year-old and 33-year-old stands (*p* < 0.05). Moreover, significant relationships were found among the activity of C-, N-, and P-acquiring enzymes (Figure 3).

### 3.3. Vector-Based Characteristics of Extracellular Enzyme Stoichiometry

**Figure 4:**
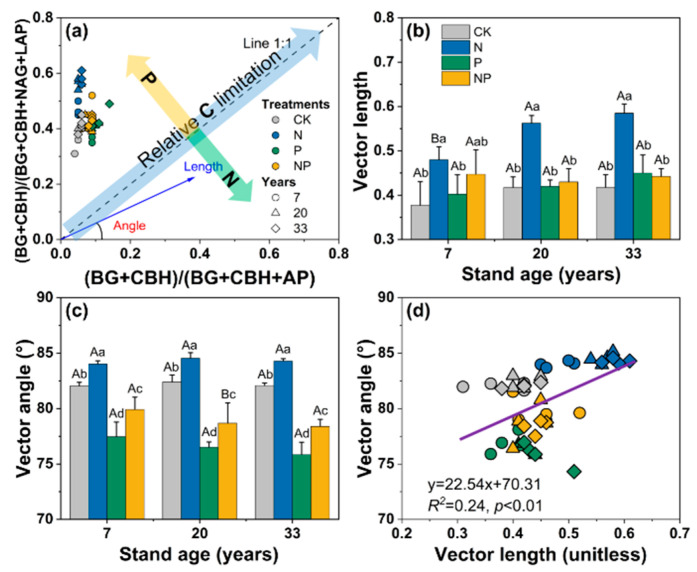
Stoichiometry of the relative proportions of the enzymatic acquisition of C and N versus the enzymatic acquisition of C and P (**a**). Variations in vector length and angle (**b**,**c**) and their relationships (**d**). Values are the means ± standard error (*n* = 4). Different lowercase letters mean significant differences (*p* < 0.05) among different treatments in stands of the same age, and different uppercase letters mean significant differences among different stand ages under the same treatment. The linear regression in (**d**) was to identify the relationships of microbial C limitations with microbial N/P limitations.

The characteristics of enzymatic stoichiometry differed among the nutrient treatments and stand ages (Figure 4a). All data points were above the 1:1 line, indicating a strong P limitation in the soil microbial community. N addition significantly increased vector length (microbial C limitation) and vector angle (microbial P limitation) in stands of all ages (*p* < 0.01, Figure 4b,c). Both P and NP addition had no significant effects on vector length but largely decreased vector angle in all stand ages (*p* < 0.01, Figure 4c). Vector lengths were larger for 20-year-old and 33-year-old stands than for the 7-year-old stand under the addition of N, and vector angles were smaller under the P treatment compared with the NP treatment. The vector angles were all >45°, indicating that microbial metabolism was limited by soil P. In addition, microbial C limitation was significantly correlated with microbial P limitation (Figure 4d).

### 3.4. Correlation of Enzymatic Activity and Stoichiometry with the Soil’s Properties and Microbial Structure

**Figure 5:**
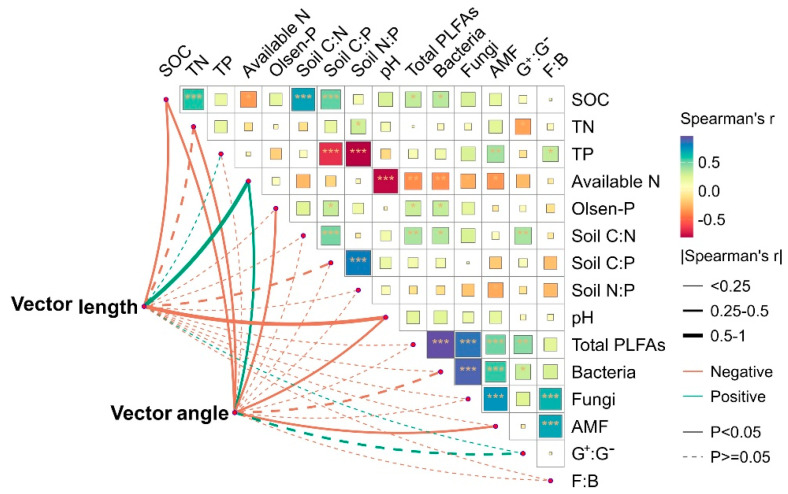
Spearman’s correlations (r) of vector length and angle with the properties of the soil and microbes. The thicknesses of the lines indicate the strength of the correlation, and the asterisks indicate statistical significance (* *p* < 0.05, ** *p* < 0.01, *** *p* < 0.001).

**Figure 6:**
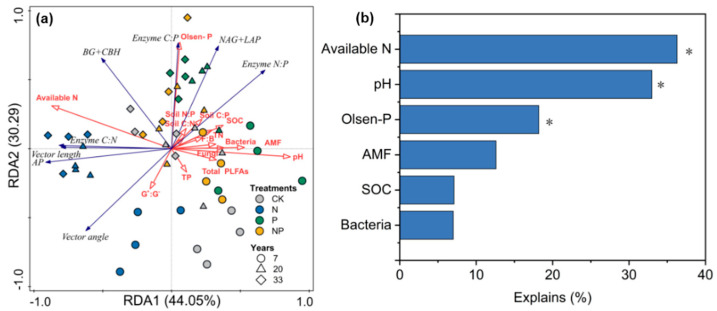
Redundancy analysis (RDA) was used to identify the relationships among the soil enzymes’ stoichiometry (blue arrow) and abiotic and biotic factors (red arrow) (**a**). The main factors contributing to the explanation are shown in (**b**). * *p* < 0.05.

The results of the Spearman’s correlation and the Mantel test heatmap showed that vector length was highly significantly positively correlated with SOC and pH, and negatively correlated with soil-available N (the sum of NH_4_^+^-N and NO_3_^−^-N) (Figure 5). Vector angle was significantly positively correlated with SOC, TN, Olsen-P, pH, and AMF, and negatively correlated with soil-available N (Figure 5). The RDA results showed that the first axis explained 44.05% of the variables, and the second axis explained 30.29% of the variables (Figure 6a). Soil-available N (36.3%, *F* = 26.2, *p* = 0.03), pH (33%; *F* = 22.6, *p* = 0.03), and Olsen-P (18.2%; *F* = 10.2, *p* = 0.03) had the longest arrows, indicating that in comparison with other factors, the soil’s N and P availability better explained the changes in soil enzyme activity and the stoichiometric ratio (Figure 6b).

## 4. Discussion

### 4.1. Effect of N Addition on Soil Enzyme Activity and Stoichiometry

Our results showed that N addition had significant effects on the soil’s EEAs in Chinese fir plantations of different stand ages. Generally, the addition of N increased the activity of BG + CBH, decreased that of NAG + LAP, and increased that of AP (Figure 3), indicating that these enzymes are easily stimulated by the N substrate. This result supports the resource allocation theory of enzymatic production [39], which predicts that N addition increases the activities of C- and P-cycling enzymes but suppresses the activity of N-cycling enzymes. Many pieces of evidence have revealed that soil microbial communities and activities are sensitive to increasing N and P contents [6]. It was found that BG, NAG + LAP, and AP activities showed a gradual increase with an increase in the age of the Chinese fir plantation, which is consistent with previous reports [40]. This may be due to the different structures of the stands at different ages, and changes in the stands’ structures may have caused changes in the microbial species and their composition, which, in turn, would affect soil enzyme activities [41]. As the stand develops, the understory vegetation layer develops and nutrients are elevated, and the active substances released by microbial metabolism increase, promoting microbial reproduction and activity, thus leading to higher enzymatic activities [42].

N addition significantly increased the soil’s available N content, while pH was significantly lower compared to the control (Figure 1). pH was a significant factor affecting soil enzyme activity and the enzymes’ stoichiometric ratio. VL was significantly negatively correlated with pH, and VA was significantly positively correlated with soil-available N. This indicated that the N treatment changed the chemical properties of the soil and thus caused changes in soil enzyme activity. It has been shown that pH can either directly promote or inhibit enzymatic catalysis or indirectly affect enzyme activity by influencing microbial proliferation [43]. A meta-analysis indicated that changes induced in soil pH are a key driver of the impact of global change on soil microbiology and N cycling [44]. Soil pH can influence microbial physiology, substrate–enzyme binding, and the formation of enzymatic proteins [45]. N addition induced soil acidification, which has been frequently observed in subtropical soils due to the leaching of magnesium and calcium and the mobilization of aluminum [15,46]. Moreover, the increased nitrification and consumption of NH_4_^+^ by plants’ roots and soil microorganisms induced by N addition resulted in the release of more H^+^ into the soil [47].

The enzymes’ C:P and N:P ratios were observed to be less than 1 and lower than the C:N ratio (Figure 3). Across all stand ages, the enzymes’ C:N ratios were significantly higher under the N treatment, while the enzymes’ N:P ratios were significantly lower compared with the control. VA was greater than 45° across treatments and stand ages, with all data points above the 1:1 line (Figure 4), indicating that soil microorganisms in Chinese fir plantations in this area were P-limited. N addition significantly increased VL and VA (Figure 4), suggesting that it exacerbates soil C and P limitations, which is consistent with previous studies [48]. This study further provides valid evidence that the addition of N exacerbates P limitations in terms of soil enzyme activity and stoichiometric ratios. In the Chinese fir stands that we studied, aboveground tree growth after 3 years of treatment showed a weak positive response to N addition, with an increase of 7.8% in diameter. The increased tree growth and the uptake of soil nutrients would exacerbate the lack of P content in the soil. When soil P levels are low, soil microorganisms or plants will continue to obtain effective soil P by adjusting their allocation of biomass and their physiological and biochemical reactions. The lack of soil P will also promote the release of more AP enzymes by microorganisms or plants to increase the availability of P. N is an important element in the composition of phosphatase proteins, and N addition facilitates the synthesis of P-acquiring enzymes [49]. On the other hand, the N treatment increased the total content of PLFAs, bacterial, fungi, AMF, and the F:B ratio, especially in the 7-year-old stand (Figure 2). These results suggest that the addition of N exacerbated P limitations in the region for three reasons. First, unlike N, soil P is derived from the weathering of rock, and each ecosystem has a relatively fixed amount of P reserves. The abundance of precipitation in the subtropics and the development of ecosystems may result in a continuous loss of soil P that cannot be easily replenished [27,50]. Second, N addition increases microbial abundance and the demand for P, and also promotes plant growth and increases the use of P in plants, making it difficult to maintain the effectiveness of soil P [8]. Lastly, a survey of the understory vegetation found that N addition reduced the abundance of understory vegetation [51], which may increase the loss of soil P due to erosion in the long term [52]. The results of this study may provide a reference for cycling soil nutrients and management practices in subtropical fir plantations in the context of N deposition.

### 4.2. Effect of P Addition on Soil Enzyme Activity and Stoichiometry

This study found that P or NP addition significantly reduced the activity of soil AP in stands of all ages (Figure 3), which is consistent with previous studies [4,53]. Furthermore, significant increases in the enzymes’ C:P and N:P ratios were observed under the P treatments at all stand ages (Figure 3), suggesting that the application of P alleviates plant and microbial P limitations. Many studies have suggested that long-term exogenous P inputs reduce the microbial demand for P, which, in turn, reduces the microbial secretion of phosphatase and inhibits the activity of AP [54]. Our results indicated that AP activity was reduced under the addition of P, either because the application of P directly inhibited the secretion of phosphatase by the soil microorganisms or plant roots, by reducing the demand for P by soil microorganisms, or by reducing the energy input for the synthesis of phosphatase by microorganisms [54,55]. According to the principle of evolutionary economics, microorganisms reduce their metabolic investment in the production of phosphatase when sufficient inorganic P is available [56]. The RDA results indicated that Olsen-P was the main influencing factor affecting enzymatic activity and enzymes’ stoichiometric characteristics (Figure 6). This is similar to the results of other studies on subtropical forests [57,58,59], where the application of P eases the competition for P between plants and microorganisms, and thus they invest less in the release of AP. This mechanism is often referred to as “end-product suppression” and is often present in soils where available P is added [57,60].

N deposition generally causes forest ecosystems to shift from N to P limitations [61] and can further exacerbate plants’ P limitations [62]; thus, P application may mitigate the effects of N deposition on plants and alleviate plant and microbial P limitations. Our results validated this hypothesis, with a significant reduction in vector angle under the P treatment compared with the control in all stand ages (Figure 4). In addition, the vector analysis showed no significant change in vector length under the P treatment compared with the control in all stand ages, indicating that the addition of P had no significant effect on microbial C demand. The source of AP enzymes is very complicated and may be related to soil microorganisms and plant and root secretions. This reflects the complexity of enzyme and nutrient limitations [21], which need to be further explored.

### 4.3. Implications for Plantation Management

Our study provides evidence that soil enzyme activities and stoichiometry can be applied to interpret the changes in microbial C nutrient limitations resulting from exogenous inputs. N and P addition provide exogenous nutrients to soil microorganisms, causing changes in the C:N:P ratio that likely affect microbial strategies for acquiring resources [8,63]. The microbial response to N deposition and P addition, which is responsible for soil C acquisition, will be useful in predicting ecosystems’ resilience to future global changes. Moreover, it is believed that microorganisms have a competitive advantage over plants in obtaining nutrients from the soil, and if microorganisms are limited by a nutrient, then plants should also be limited by that nutrient [64]. Our previous study of aboveground nutrient reabsorption indicated that the nutrient demand in Chinese fir plantations is characterized by a shift from the co-limitation of N and P in young stands to P limitation in mature stands [28]. Thus, the study of limitations in soil microbial resources should help develop management strategies to control soil C and nutrient cycling, such as through P fertilization, to improve a plantation’s productivity despite increasing N deposition. Future studies should investigate the impacts of nutrient addition on other soil biogeochemical cycles or explore the potential interactions between N deposition and other environmental stressors (e.g., drought and warming) for the better management of plantations.

## 5. Conclusions

In the present study, the effects of adding N and P over 3 years on soil enzyme activity and stoichiometry were examined in Chinese fir plantations of different stand ages. The results showed that N addition alone increased the activity of BG + CBH, enzymatic C:N ratios, and the activity of AP, and decreased the activity of NAG + LAP and enzymes’ N:P ratio, thereby aggravating microbial C and P limitations (i.e., both vector length and angle increased). Both P and NP addition alleviated microbial P limitation, as revealed by the significant decrease in vector angle and the enzymatic N:P ratio. Soil-available N, Olsen-P, and pH were the main factors influencing enzyme activity and stoichiometry overall. These results indicate that changes in soil nutrients’ availability induced by N and P inputs influence the composition and activity of soil microbes and lead to changes in the microbes’ strategies for acquiring resources. Our study further suggests that P fertilization in subtropical plantations may be an effective method of maintaining productivity under the scenario of the increased deposition of N. This study provides useful insights into changes in the limitations of soil microbial resources under exogenous N and P inputs, and provides a reference for determining effective fertilization strategies to improve planted forests’ productivity and soil C sinks.

## Data Availability

The original data generated in this study are included in this article. Further enquiries can be directed to the corresponding author.
